# Fiji: Its Hospitals and Diseases

**Published:** 1908-04-04

**Authors:** T. R. St. Johnston

**Affiliations:** Resident Medical Superintendent, Colonial Hospital, Suva.


### HOSPITAL ADMINISTRATION.

### CONSTRUCTION AND ECONOMICS.

### FIJI: ITS HOSPITALS AND DISEASES.—I.

By T. R. St. JOHNSTON, M.R.C.S., L.R.C.P., M.S.A., Resident Medical Superintendent, Colonial Hospital, Suva.

A hospital in the Fiji Islands! An up-to-date, modern British hospital and a school of medicine for students; these things are somewhat surprising at first to those who land in Fiji with preconceived notions and somewhat hazy ideas about the so-called "Cannibal Islands."

Fiji has a strange and little-known history, stranger, perhaps, than any other of the British Colonies, and, being hidden away in the farthest corner of the whole world, it is even now, in this modern century of tourists and trippers, so little talked of and so seldom written about that anything relating to it may have at least the merit of freshness. Only a year or two ago a large British trading vessel was wrecked off the coast, and, incredible as it may seem, the crew of some fifty men were rowing about for four days, afraid to land because they imagined Fiji to be still full of cannibals. Fiji, with its telephones, its free libraries, its policemen! But so it is. The country is too far from England to be visited except by those with the longest purses; it is out of the track of the newspaper reporter and popular magazine writer, and the result is a strange and novel place, a paradise for the *blasé* and ennui-stricken, and a little corner of the earth's surface without a music-hall and a daily newspaper. But if it sighs in vain for these latter products of civilisation, it has a more stable sign of a real and rapid advancement in the large and well-found hospital at Suva, the capital.

This hospital, in company with most other public buildings in the islands, is a Government institution and entirely staffed by Government officials; but in all the main points it is conducted in a manner very little different to an ordinary general hospital in England. The underlying principles are just the same, and it is only at first sight that it seems so strange to people fresh from home. But these superficial differences are so novel and so interesting that a description of them may be worth attention.

The hospital itself is built on a little hill in a beautiful situation commanding the bay, and, although a mile from the town, there is a splendid carriage road the whole way, so that accidents and urgent cases do not suffer from the distance, while the benefit of the fresh sea-breezes that are always present is of inestimable value. On approaching the hospital the first thing that usually attracts one's notice is a little group of natives sitting on their heels in a compound under the shadow of a large bread-fruit tree. These are the out-patients and ordinary cases awaiting admission.

One may say at once that the native patients treated here are of many races—Fijians, Samoans, wild-looking men of the New Hebrides and Solomon Islands, Hindoos, Mussulmans, and occasional Chinamen and Japanese, so that to those fresh from England the language question is a very real and ever-present difficulty. These different races, too, create the necessity of a number of different blocks or one-story buildings, so that the whole hospital is really built on the pavilion system. In former years Fijians and Indians, for instance, would never reside amicably under the same roof, and there were frequent squabbles and petty fights; but latterly they have, through constant association, become more indifferent to one another's failings.

In the main block are the offices, dispensary, operating-theatre, out-patient room, etc., and the principal wards for European patients; while close at hand are smaller detached private wards, each containing only one or two beds, also for European patients. If we go round this block we shall probably find much the same patients as in an English hospital, cases of a medical, surgical, or gynæcological nature; but in addition we are sure to see one or two instances of tropical disease, such as dengue, dysentery, or hepatic abscess, and, more rarely, elephantiasis and yaws. Apart from these, the only novel thing that strikes the eye is the necessary absence of heavy bed-clothes, often a single sheet being all-sufficient, and the presence of mosquito-curtains on all the bedsteads.

But it is with the native wards that we are most interested. On first entering them one is struck by the apparent bareness and emptiness of them, though they are probably—and usually—full of patients. This is entirely due to the beds, which are devoid of the mattresses, pillows, and white counterpanes one usually associates with a bedstead in England. And until one misses them one cannot realise how these things help to furnish a ward. The first impression, then, is how uncomfortable these poor patients must be! Far from it. All these things have been tried many times; but the Eastern mind thinks differently, and it was invariably found in the morning that these so-called luxuries were carefully rolled up and deposited under the bed, and the patient seen lying back, smiling and happy, on a bare iron mattress with his own native mat wrapped around him. At first they would not even have the bedsteads, and insisted on lying on the floor; but after a time their suspicion of these strange English contrivances died away, but the bed-clothes are still too much for them, and they are really more comfortable in their mats.

Plain grey rugs are supplied to all the native patients, and the Indians usually prefer these and make use of them; but the Fijians and Polynesians keep either to mats beautifully woven from small dried grasses or to rugs made of the native tappa, that wonderful cloth that is beaten out with heavy mallets from the bark of a tree and dyed with many strange coloured devices.

Then again, the positions of the patients seem at first strange. All the Fijians and Polynesians are seen squatting cross-legged on the top of their beds, for all the world like long rows of tailors, and only when asleep or when desperately ill will they be found in any other position. This is their attitude of rest, and in the women's wards the same curious position is seen.

The daily round is done in the morning, and is much the same as a visit in a teaching hospital at home, except that the medical students are, to say the least of it, somewhat surprising in appearance. Imagine about a dozen or fourteen tall muscular fellows of splendid physique and of a dark copper colour, attired in only a white tennis shirt and a short petticoat reaching to the knees, and surmounted by an enormous mass of stiff straight hair, often six or eight inches in height, and you have the medical students of Fiji. But such fine young men—polite, good-natured, quick, and willing to learn, and often possessing keen clinical powers and medical skill which would excel many students of their own year at home if given similar opportunities of study.

These students are usually selected from the better-class and princely families, and their good lineage is shown in their regular and handsome features and superior intelligence; but the selection of students is taken solely upon merit, and everyone has a right to compete in the examinations which are held. There is never any lack of applicants, and they usually enter at about the age of 16 or 17, after passing a preliminary examination in general education. Though English is not compulsory they can usually speak a little, and understand more; but in their own language they are all well educated, a high standard being kept up in the native schools.

One of the visitors of the Marist Native School only last week reported, in reference to the Fijian pupils: "It was simply astonishing to hear these boys, whose average age was between eight and nine years, reading and understanding English, Hindustani, and Fijian, and rattling off clever answers to all manner of questions with ease and confidence, and displaying work in the shape of splendid maps and all kinds of clever pen and pencil efforts."

The students are divided into first, second, and third year classes, and at the expiration of their time have a final qualifying examination which gives them a diploma in medicine recognised by the Colony, and they take the title of "native medical practitioner"; mostly being employed in the Government service. As amongst students all over the world, there are some who are lazy and some who are stupid, and the pass-list is just as anxiously awaited out in Fiji as at Lincoln's Inn Fields at home, especially as here they are only allowed three failures.

There are also one or two Fijian and Indian hospital attendants, but all the real nursing is done by a staff of English nurses, and probationers are also trained and take out their hospital lectures and examinations here.

But to return to the patients. It is interesting to contrast the temperaments of the three great classes of natives that are treated here—Fijians, Indians, and Polynesians. The Fijians—stalwart handsome men, with the kindest and gentlest of voices and manners—are intelligent enough to appreciate the value of coming to hospital, and are the best type of patient, the type that has faith in the ultimate result of their treatment. But for all that they are a race that is dying away—more the pity—and every new white man's ailment they incur sweeps through them with tenfold virulence. At one time it was measles, and in one black year this apparently trivial disease killed one-third of the total population. Now phthisis has them in its grip, and despite all precautions there is still a high mortality from this. It has been seriously said that in forty years Fiji will not possess a Fijian, but will be totally inhabited by Indians. This may be an exaggeration; but it is a fact that the Indian labourers, who are pouring into the plantations every year, are both fertile and wiry, and though they seem to incur a larger proportion of what in England we should call the serious diseases, yet the so-called trivial ailments are fortunately to them only trivial.

These Indians are clever and cunning, cringing little men, and inveterate thieves. We have, of course, only the lower castes, who come as indentured labourers for a term of years, and they are not pleasant people to have dealings with. From a hospital point of view they are in many respects closely allied to certain hospital out-patients in London, in that they are dirty, come up to the hospitals for free treatment for every trifling disorder, and are regular "hospital birds" in their constant attempts to get board and lodging as in-patients by malingering.

The Polynesians are little sturdy fellows from all the other South Sea Islands—the Solomon, New Hebrides, New Guinea, and Gilbert Islands supplying most of their number. Many also have served for years in Queensland on the plantations, and, having been driven out by the Australian "anti-colour" legislation, have found their way to Fiji, being afraid to go back to their own islands in the New Hebrides or Solomon groups. Cannibalism, if questioned, still flourishes in those islands, though they always deny it, and from their long absence these men would be regarded as foreigners by their fellow-tribesmen. These little Polynesians are childish in their ways and irritable and fractious as patients, and if not improving in health often say they are going to die, and resolutely setting their minds on this they often do die, where a white man would get well.

The natives of these other South-Sea groups speak a totally different language to Fijian, and again, men from two small neighbouring islands will often not even understand each other, so that the hospital officials find it impossible to master the hundred and one languages which exist in the South Seas, and have to be content with occasional smatterings of French, German, Italian, etc., as wanderers from other parts of the world are drifted in. The result is that the Polynesians have to pick up a sort of pidgin-English, and very funny it sometimes is. It is for instance, somewhat disconcerting when asking how a patient with rheumatism is to have him point to first one leg and then the other, and say: "Dis fellow plenty sick, all same along dis fellow. Me pever (fever) no finish, I tink me b'long die."

Quaint-looking men they are, too, with broad, flat noses, short curly hair, and little squat figures. Fierce-looking blue tattoo marks are seen round both eyes, the lobes of their ears are pierced, and the holes distended by discs of wood, often measuring two inches across, while about a dozen small pieces of wood, like broken-off matches, are inserted along the margin of the alar nasæ. The average Fijian, with his sulu or petticoat, I have already described in mentioning the medical students; and the Indians, with their turbans and dhotis, are known to everyone, either by personal experience or through pictures.

